# Analysis of palatal rugae pattern and maxillary sinus index for gender determination

**DOI:** 10.6026/973206300200748

**Published:** 2024-07-31

**Authors:** Himalee Mehta, Saurabh Goel, Kavita Verma, Barkha Makhijani, Meet Mehta, Shivani Maheshwari, Adarsh Dixit

**Affiliations:** 1 Department of Oral Medicine and Radiology, Pacific Dental College and Research Centre, Bhilo Ka Bedla, Udaipur, Rajasthan, India; 2 Department of Periodontics, Pacific Dental College and Research Centre, Bhilo Ka Bedla, Udaipur, Rajasthan, India; 3 Department of Oral and Maxillofacial Surgery, Nathalal hospital. Rajkot Gujarat, Rajkot, Gujarat, India; 4 Department of Oral Medicine and Radiology, Sardar Patel Post Graduate Institute of Dental and Medical Sciences, Lucknow, Uttar Pradesh, India

**Keywords:** Forensic dentistry, rugoscopy, lateral cephalometric radiographic technique, gender determination

## Abstract

Correlation between rugoscopy and lateral cephalometric radiographic technique for gender determination is of interest. A cross sectional study was conducted on 100 subjects within an age group of 20 to 50 years. Distribution of rugae patterns and morphometric analysis of maxillary sinus was done for gender correlation. Wavy curved and straight rugae patterns were observed to be more in female gender as compared to males. The mean MSI was higher in females (1.32) when compared with males (1.26). Both the morphometric analysis of maxillary sinus and rugoscopy has been proved to be a valuable tool in the assessment of sexual dimorphism. But, morphometric analysis of maxillary sinus is relatively a new and reliable method for gender determination using maxillary sinus index.

## Background:

Identity of a person does not end at his finger, palm, or foot prints but also includes his distinct dental features from a forensic point of view [1]. The customary methods for human identification include anthropometry, fingerprints, dental records, gender determination, age estimation, weighing, and identification by specific characteristics, and blood-group differentiation [2]. Gender can be determined by various methodologies such as sexual dimorphism with tooth morphology, pulpal DNA analysis, and study of lip prints, palatal rugae, and finger prints and even with radiological techniques by morphometric analysis of paranasal sinuses [3]. Palatal rugae is defined as an anatomical fold or wrinkle usually made in the plural sense; the irregular fibrous connective tissue ridges located in the anterior third of the hard palate. They appear in the 3rd month of intra-uterine life. Due to its anatomic position, rugae are protected from thermal insults by the tongue and buccal pad of fat [4]. Rugoscopy involves the study of palatal rugae pattern for human identification where finger prints are unavailable. As palatal rugae patterns are genetically determined, they can also be used in population differentiation and gender determination. The study of anthropometric characteristics is of fundamental importance to solve problems related to human identification. Among the human bones, next to the pelvis, the skull is the most easily sexed portion of the skeleton. In cases of mass disasters, even the skull and other bones are badly blemished; however, maxillary sinuses remain intact [5]. Sinus radiography has been used for identification of skeletal remains and determination of gender. Lateral cephalogram plays a predominant role providing architectural and morphological details of the skull, thereby revealing supplementary characteristics and multiple points for comparison .Various researchers have alleged this conventional radiograph as cost effective, easily available, easy to perform, offers quick results, reproducible and easily implemented and reliable in providing accuracy of 80-100% [6]. Therefore, it is of interest to compare & correlate between Rugoscopy and Lateral Cephalometric Radiographic technique for gender determination.

## Materials and Methods:

Randomized controlled single blind cross sectional study had been conducted in the Department of the Oral Medicine and Radiology, Pacific Dental College and Research Centre, Bedla, Udaipur. In this study 100 Patients who had been willing to participate in the study were selected randomly (male & female) between the age group of 20 to 50 years Udaipur after obtaining ethical clearance from the ethical committee. An informed consent had been taken for all the patients. Subjects with Facial trauma/ Pathology including maxilla & maxillary sinus, Congenital Anomalies, Endocrinal disorders, Nutritional disturbances , Previous History or undergoing Orthodontic treatment and Pregnant women were excluded from the study. Maxillary cast prepared was for the study of Palatal Rugae patterns and Lateral Cephalogram for the measurement of Maxillary Sinus of the same patient was recorded. Firstly, measurement of the rugae was analyzed as per Kapali *et al.* (1997) [7], Thomas and Kotze 1983 [8] classification later, the digital lateral Cephalogram (Carestream dental CS 8100) was taken followed by tracing and measurement of maxillary sinus height and width. Maxillary sinus index (MSI) was calculated as follows: MSI = maxillary sinus width/height.

## Results:

Amongst males there were 26 (52%), 11 (22%), 4 (8%), 9 (18%) , 16 (32%) and 34 (68%) wavy straight, curved circular and convergent, divergent respectively while in females there were 28 (56%),14 (28%), 5 (1o%), 3 (6%), 7 (14%) and 43 (86%) rugae in respective groups. 31-40 years old study subjects were found to have more wavy and curved rugae, 21-30 and 41-50 years were found to have more straight rugae circular rugae respectively. On applying chi square test results were observed to be statistically significant (χ^2^ = 494.742, p= 0.000, S.) There was an insignificant (p>0.05) gender differences in the pattern of rugae among male and female students except straight and circular pattern which showed a significant difference between both gender. On morphometric analysis of maxillary sinus, the mean maxillary sinus height was found to be 28.4 mm in males and 26.5 mm in females and it was statistically significant with (0.5648 to 3.122) 95% CI and p value of 0.0056. The mean maxillary sinus width was 36.1 mm in males and 35.1 mm in females which was statistically non-significant with (-0.8230 to 2.377) 95% CI and p value of 0.3244. The mean MSI was higher in females (1.32) when compared with males (1.26) with (-0.1486 to -0.01389) 95% CI and a significant p value of 0.0202. Discriminant analysis was done using gender as a grouping variable and MSI as an independent variable, the obtained determinant equation when applied to the study sample revealed that 36 out of 50 were correctly identified as males and 34 out of 5o as females with sensitivity of 72% and specificity of 68%.

## Discussion:

Considerable amount of literature is present on genetic predisposition, prevalence and morphological patterns of rugae [9,10]. To date, there is scarce literature present on whether sex can be predicted through palatal rugae patterns. Zehra *et al.* 2022[11] showed higher number of rugae in males as compared to females and frequency of straight, curved and wavy are significantly higher in males than in females wherein our study frequency of straight, curved and wavy rugae patterns were higher in females. Present study indicated an association between rugae pattern and different gender groups of different population. Hence, palatal rugae are specific for the male and female population, and have a possible role in identifying gender. Though the study indicated the use of palatal rugae as an adjuvant in sex identification in a small sample size confirmation of the results over a larger population size is required involving different ethnic groups are required to explore the potential of palatal rugae patterns in forensic dentistry. Morphometric analysis of paranasal sinuses was done to determine gender. The mean MSI was higher in females and the lowest value being presented by MSI, indicating MSI to be comparatively a better indicator for sex determination among all the variables. When Discriminant analysis was done, 36 out of 5o were correctly identified as males and 34 out of 5o as females with sensitivity of 72% and specificity of 68%. Chandra *et al.* (2014) [12] established the accuracy and reliability of maxillary sinus in gender determination using morphometric parameters (area and perimeter), using lateral cephalogram. The correct predictive accuracy was found to be 70.8% in males and 62.5% in females. Teke *et al.* (2007) [13] established the accuracy of gender determination of 69.4% in females and 69.2% in males. Uthman *et al.* (2011) [14] concluded that 74.4% of male sinuses and 73.3% of female sinuses were sexed correctly and the overall percentage for sexing maxillary sinuses correctly was 73.9%.

## Conclusion:

Morphometric analysis of maxillary sinus and rugoscopy has been proved to be a valuable tool in the assessment of sexual dimorphism. But, morphometric analysis of maxillary sinus is relatively a new and reliable method for gender determination using maxillary sinus index. However, further studies are desirable on large sample size.

## Financial support:

NIL
